# Effect of Defects in Graphene/Cu Composites on the Density of States

**DOI:** 10.3390/ma16030962

**Published:** 2023-01-20

**Authors:** Song Mi Kim, Woo Rim Park, Jun Seok Park, Sang Min Song, Oh Heon Kwon

**Affiliations:** 1 Department of Safety Engineering, Graduate School, Pukyong National University, Busan 48513, Republic of Korea; 2 Korea Industrial Safety Association, Seoul 08289, Republic of Korea; 3 Samsung Electronics Co., Ltd., Suwon 16677, Republic of Korea; 4 Department of Safety Engineering, Pukyong National University, Busan 48513, Republic of Korea

**Keywords:** defect, DFT (density functional theory), DOS (density of states), Fermi level, graphene/Cu composites

## Abstract

The process of handling and bonding copper (Cu) and graphene inevitably creates defects. To use graphene/Cu composites as electronic devices with new physical properties, it is essential to evaluate the effect of such defects. Since graphene is an ultrathin anisotropic material having a hexagonal structure, an evaluation of graphene/Cu composites containing defects was conducted taking into account the inherent structural characteristics. The purpose of this study is to evaluate defects that may occur in the manufacturing process and to present a usable basic method for the stable design research and development of copper/graphene composites essential for commercialization of copper/graphene composites. In the future, when performing analytical calculations on various copper/graphene composites and defect shapes in addition to the defect conditions presented in this paper, it is considered that it can be used as a useful method considering defects that occur during application to products of desired thickness and size. Herein, density functional theory was used to evaluate the behavior of graphene/Cu composites containing defects. The density of states (DOS) values were also calculated. The analysis was implemented using three kinds of models comprising defect-free graphene and two- and four-layered graphene/Cu composites containing defects. DOS and Fermi energy levels were used to gage the effect of defects on electrical properties.

## 1. Introduction

Graphene is a two-dimensional nanomaterial with electron mobility of 15,000 cm^2^·V^−1^·s^−1^ at room temperature, resistivity of 10^−6^ Ω·cm, carbon–carbon bond length of 0.142 nm, and interlayer separation of 0.33 nm [1,2,3]. Using graphene in a composite may provide nanodevices with scalability. Many studies have examined graphene composited with copper (Cu), which has excellent electrical conductivity [4]. For example, Dong et al. [5] studied the electrical conductivity and strength of graphene/Cu nanofilms, Wu et al. [6] analyzed the electrochemical properties of graphene/metal composites for use as energy storage devices, and Jiang et al. [7] compared graphene transistors, silicon transistors, and graphene varistors.

However, defects due to the handling of ultrathin graphene inevitably occur when graphene is composited with Cu. Defects that occur during graphene manufacture make it difficult to identify physical mechanisms related to electrical properties. Therefore, to use graphene/Cu composites in a device, it is necessary to evaluate the effect of defects in the graphene/Cu structure while considering the number of laminated layers. Boukhvalov et al. [8] studied the stability of the graphene/Cu surface using density functional theory (DFT) modeling. This theory enables the calculation of multibody systems by viewing electrons as a single set of electron densities. Garcia-Rodrigez et al. [9] conducted a DFT study of Cu nanoparticles adsorbed on defective graphene. Aslanidou et al. [10] generated fewer than ten layers of graphene bonded to electronic devices via chemical vapor deposition (CVD), and Sherrell et al. [11] studied the electrical stimulation of graphene produced by CVD. Gallegos et al. [12] calculate using DFT to state magnetic and electronic changes of Mn in ZnO and Maleki-Ghaleh [13] et al. investigated the characterization and optical properties of nanoparticles and electronic structure by DFT.

To evaluate the effect of defects on the electrical behavior of graphene/Cu composites at the atomic and molecular levels, this study analyzed the electrical properties of the composites and physical mechanisms of defects. The density of states (DOS) relates to electron occupation at a specific energy level, and DOS value indicates the electrical behavior caused by the movement of free electrons (by knowing the energy level at which electrons or holes may exist). The aim of this study is to figure out the effect of defects present in graphene/Cu composites using DFT analysis method. The tool we used is Quantum Espresso which is an open-source software for nanoscale electronic structure calculations and material modeling. The results of this study provide a basis to evaluate the effect of defects on the electrical behavior of graphene/Cu composites.

## 2. Methodology and Modeling

### 2.1. Density Functional Theory

Density functional theory [14,15,16,17,18,19,20] is the most commonly used method in electronic structure research because it can handle electronic structures in multibody systems, such as atoms, molecules, and condensates, in the form of a single particle. It facilitates the solution of multibody systems by considering electrons as a set of electron densities, rather than considering their position as a wave function of each electron. In DFT analysis, the reliability and accuracy of the calculation can be adjusted using k-points. We used the Monkhorst–Pack approach [21], which is widely applied to converge calculations by reducing k-point error.

Density of states relates to the number of electrons that can enter a given energy level. The number of electrons that can be filled with a given energy level interval is very important for electronic and light-emitting devices whose performances are related to the flow of electrons. The Kohn–Sham equation [22,23] was used in this study as the governing equation to interpret the DFT, and is described by Equation (1) as follows:

(1) $$\left(-\frac{1}{2}\nabla^{2}+V_{eff}\left[n\right]\right)\psi_{i}\left(r\right)=\varepsilon_{i}\psi_{i}\left(r\right)$$

The Kohn–Sham equation has physical meaning, i.e., the total energy of the ground electronic state of the material system is determined only by the electron density of the ground state, and can be obtained directly by expressing the electron density in the form of a function with arbitrary spatial coordinates r for the electron’s bottom state.

The Kohn–Sham equation is the same as the single-particle Schrödinger equation for effective potential $V_{eff}\left[n\right]$, which is the orbital energy of the Kohn–Sham orbit $\varepsilon_{i}$; in turn, this is the density of the system of particle n in the Kohn–Sham state. To solve the Kohn–Sham equation, $V_{eff}\left[n\right]$ must be known, and can be calculated using Equation (2) as follows:

(2) $$V_{eff}\left[n\right]=V_{ext}\left[n\right]+\int^{\;}\frac{n\left(r^{\prime }\right)}{\left|\;r-r^{\prime }\;\right|}dr^{\prime }=v_{xc}$$

(3) $$n\left(r\right)=\overset{N}{\underset{i=1}{\sum }}{\left|\psi_{i}\left(r\right)\right|}^{2}$$

$V_{ext}\left[n\right]\;$ can be calculated using electrons close to the atomic nucleus and the potentials produced by the atomic nucleus using approximate potentials. Spatial coordinates near r are represented by $r^{\prime }\;$ and $v_{xc}\;$ corresponds to the exchange-correlation potential. To obtain $v_{xc}$, we need to know the electron density n(r), which is calculated according to Equation (3).

Obtaining the electron density by solving the Kohn–Sham equation makes it possible to reduce the experimental trial and error that may occur in the material design stage, as well as predict the density of the bottom state of the electron.

### 2.2. DFT Analysis and Modeling

DOS analysis was performed using Quantum Espresso [24] which can evaluate the nanoscale electronic structure and material modeling. Moreover, after visualizing the data obtained from the assumed modeling analysis using the material simulation platform Materials Square [25], the effect of defects in the graphene/Cu composite was analyzed. We set the k-points grid to 6 × 6 × 1. K-point grid is a calculation evaluation condition to obtain more accurate analysis results. Convergence can occur along the k-point grid. This is because it is necessary to evaluate the convergence of the integral for material properties in Brillouin zone of Monkhorst–Pack approach [21]. The higher the number of k-points used, the more accurate the analysis result, but also increases the cost. Therefore, setting an appropriate k-point is important in DOS analysis.

We use a hybrid functional method, the Perdew–Burke–Ernzerhof (PBE) series for the graphene and Cu potential conditions (Table 1) [26]. PBE increases the computational calculation time and requires a lot of memory in CPU to calculate the exchange energy, but it is a method that can improve accuracy and resolve band gap underestimation. PBE is used to obtain analysis results with adequate accuracy and efficiency. PBE also focuses on evaluating the trend of graphene/Cu composites. The cell parameters that we use are also shown in Table 1.

The analysis model was constructed for a total of eight cases as a Supplementary Information for the readers. It was based on models of individual, separate layers of graphene and Cu. The modeling of graphene/Cu composites was divided into five cases, which were further subdivided into two- and four-layer designs with and without defects (Table 2). Model 1 corresponded to single-layer graphene, and model 2 to single-layer Cu. Model 3 corresponded to the two-layered graphene/Cu composite, and model 4 to the four-layered graphene/Cu composite; this subdivision was used to evaluate the effect of the number of layers. Model 5 inserted defects into the graphene layer in the two-layered graphene/Cu composite, model 6 inserted defects only into the external graphene layer (first layer) in the four-layered graphene/Cu composite, model 7 inserted defects only into the graphene layer in the middle (third) layer, and model 8 was used to evaluate the effect of defects in the external (first) and middle (third) graphene layers.

**Figure 1:**
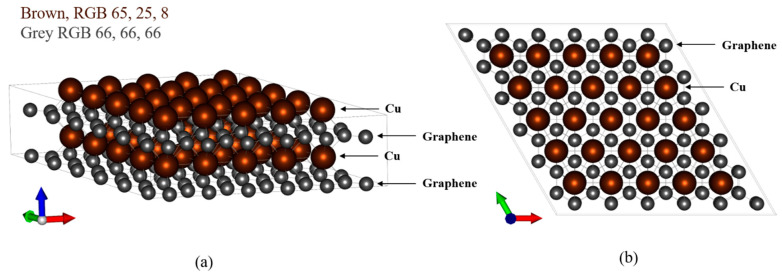
Four-layered graphene/Cu composite model (G/Cu/G/Cu). (**a**) Side view, (**b**) top view.

**Figure 2:**
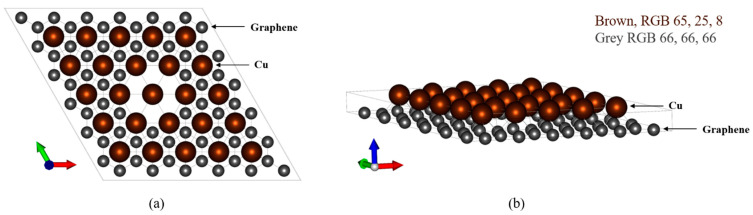
Defect positions of the two-layered graphene/Cu (G/Cu) composite model. (**a**) Side view, (**b**) top view.

The laminate was modeled according to Figure 1. In the two-layered Cu/graphene composite, the Cu layer was arranged on top of the graphene layer, and in the four-layered case, the materials were laminated in the order of graphene, Cu, graphene, and Cu. In the two-layered case, six carbon atoms were removed from the center of the graphene layer (Figure 2).

**Figure 3:**
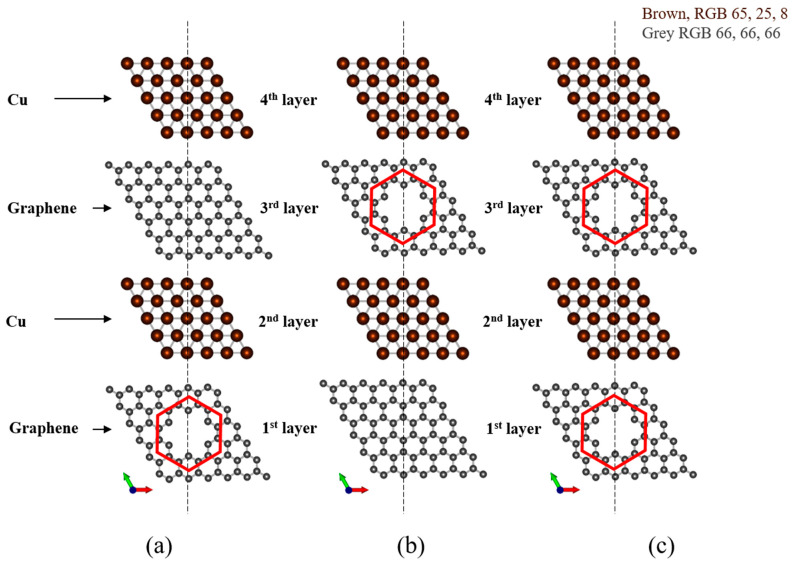
Defect positions of the four-layered Cu/graphene (G/Cu/G/Cu) composite defect model; (**a**) four layers with the defects inserted into the external graphene layer (first layer); (**b**) four layers with the defects inserted into the middle graphene layer (third layer); (**c**) four layers with defects in both the external graphene layer (first layer) and middle graphene layer (third layer).

In the case of four layers, the graphene layer into which the defects were inserted was changed. We subdivided the case to compare the following designs: four layers with the defects inserted into the external graphene layer (first layer) (Figure 3a), four layers with the defects inserted into the middle graphene layer (third layer) (Figure 3b), and four layers with the defects in both the external graphene layer (first layer), and middle graphene layer (third layer) (Figure 3c).

## 3. Results and Discussion

### 3.1. Density Functional Theory Analysis of Individual Graphene and Cu Layers

**Figure 4:**
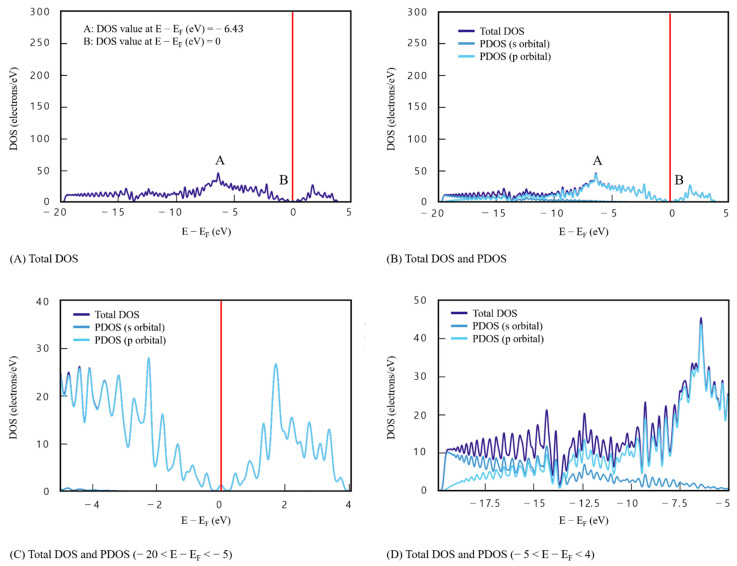
Density of states results for model 1 (single graphene layer, *x*-axis: *E* − *E_F_* (eV), *y*-axis: DOS(electrons/eV), the Fermi level is 0). (**A**) Total DOS. (**B**) Total DOS and PDOS. (**C**) Total DOS and PDOS (−20 < *E* − *E_F_* < −5). (**D**) Total DOS and PDOS (−5 < *E* − *E_F_* < 4).

The entire DOS results of monolayer graphene are presented in Figure 4A, and the PDOS results are presented in Figure 4B which were obtained via DFT analysis of monolayer graphene (model 1). The *x*-axis corresponds to the energy level function value and the *y*-axis to DOS value. DOS value of point A (the maximum) ($E_{}-E_{f}=-6.43$) is 45.36 e/eV^−1^ and DOS value of point B (the minimum) ($E_{}-E_{f}=0)$ is 0. These values indicate the intrinsic characteristics of graphene. In addition, it is evident that DOS values are relatively evenly distributed, and that the difference between points A and B is <50 e/eV^−1^. Through Figure 4C,D, it can be seen that point A is due to s orbital, and point B, which is the point where DOS becomes 0 in graphene, is due to p orbital.

**Figure 5:**
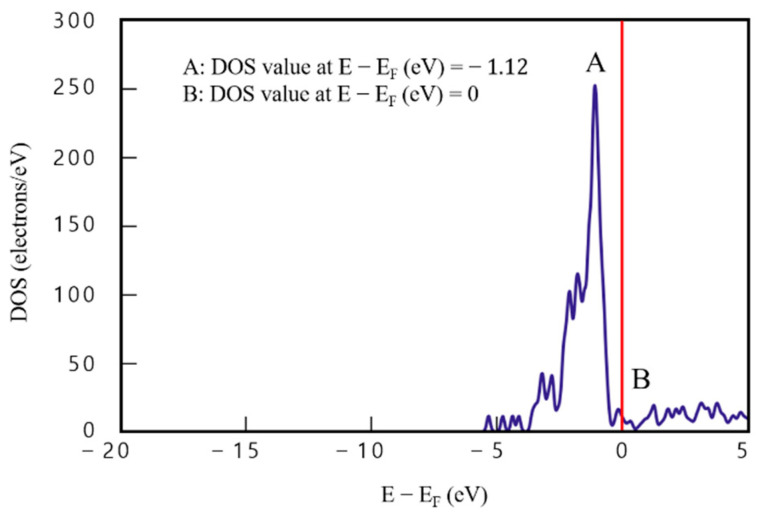
Density of states results for model 2 (single Cu layer).

DOS analysis results for the single layer of Cu (model 2) are presented in Figure 5. A high localized DOS value was observed, which is typical of metals; DOS value at point A (the maximum) ($E_{}-E_{f}=-1.12$) is 252.02 e/eV^−1^ and that at point B ($E_{}-E_{f}=0$) is 9.72 e/eV^−1^, which indicates that electrons can exist, unlike the graphene results. Moreover, the difference between points A and B is significantly larger (242.3 e/eV^−1^) compared with graphene. In addition, in model 2, DOS value was negligible before point C ($E_{}-E_{f}=-5.56$).

### 3.2. Density Functional Theory Analysis of Graphene/Cu Composites

**Figure 6:**
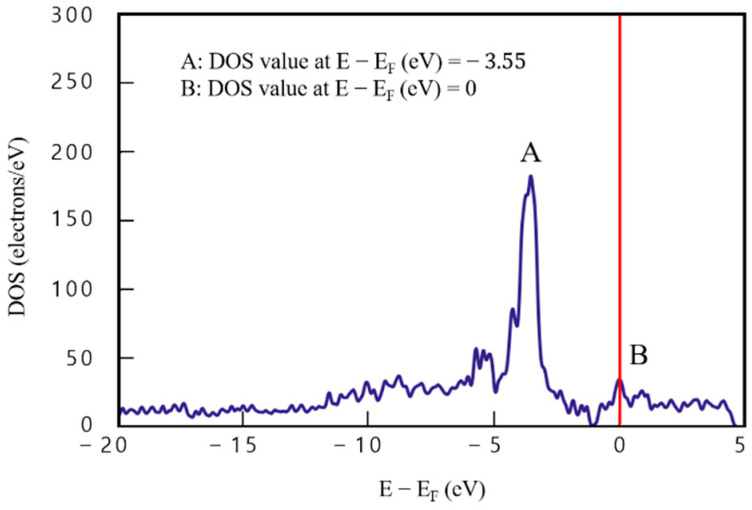
Density of states results for model 3 (two-layered graphene/Cu composite).

Figure 6 presents DOS analysis results of the two-layered graphene/Cu composite (model 3) consisting of a single layer each of Cu and graphene. In Figure 6, DOS value of point A (the maximum) ($E_{}-E_{f}=-3.55$) is 182.32 e/eV^−1^ and that of point B ($E_{}-E_{f}=0$) is 34.44 e/eV^−1^, which indicates that electrons could exist at point B, unlike in graphene. The difference between points A and B is 147.88 e/eV^−1^. In addition, DOS values are evenly distributed over the entire section ($-20<E_{}-E_{f}<5$), confirming the effect of graphene. The difference between point A, where the highest DOS value appears, and point B, where the energy level function value is 0, is approximately 148 e/eV^−1^, which is about three-times higher than that of graphene. This confirmed the effect of Cu in a section ($-3<E_{}-E_{f}<0$) between points A and B.

**Figure 7:**
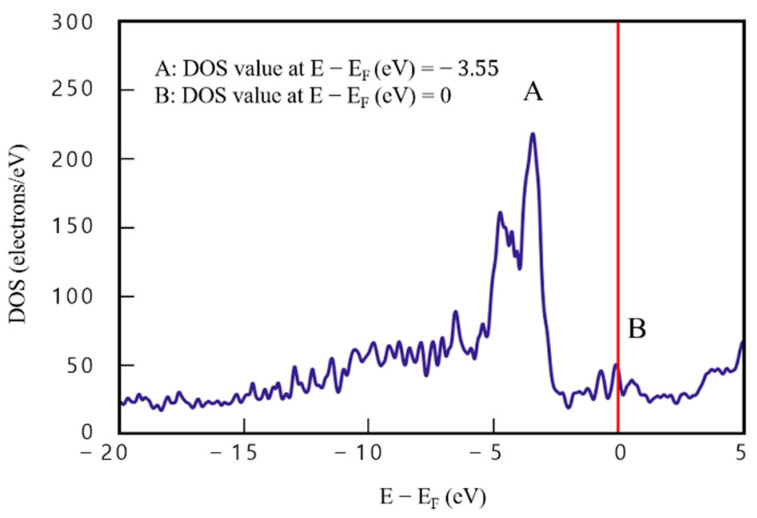
Density of states results for model 4 (four-layered graphene/Cu composite).

Figure 7 shows DOS analysis results of model 4 of the four-layered Cu/graphene composites. DOS value of 209.12 e/eV^−1^ at point A ($E_{}-E_{f}=-3.55$), which is the same point as for model 3, is 26.8 e/eV^−1^, higher than that for model 3, and DOS value at point B ($E_{}-E_{f}=0$) is 41.46 e/eV^−1^. Compared with the two-layered case, DOS value of point A is higher by about 14.7%. This finding indicates an improvement in electrical performance, because the number of electrons that can be occupied at the same energy level increased by more than 26 electrons.

In addition, DOS value was quite stable in the sections ($-20<E_{}-E_{f}<-5$) and ($3<E_{}-E_{f}<5$), and was more than double compared to that obtained with model 3. These findings indicate that the electrical performance of the graphene/Cu composites improved with increasing graphene/Cu thickness.

### 3.3. Density Functional Theory Analysis of Graphene/Cu Composites with Graphene Defects

**Figure 8:**
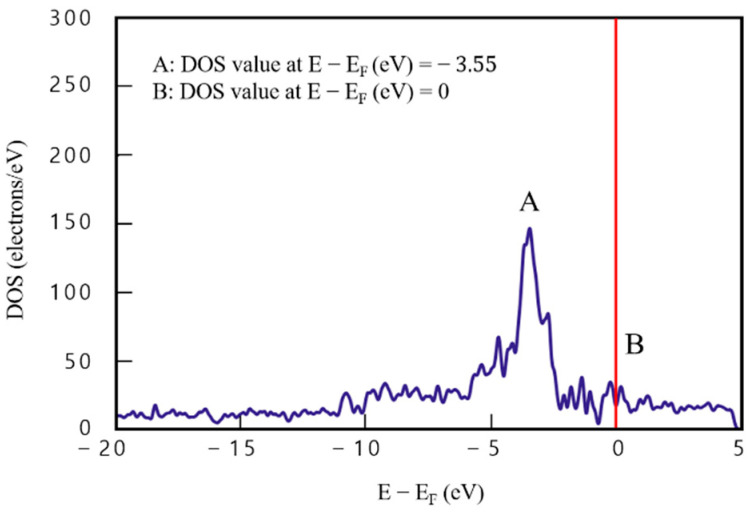
Density of states results for model 5 (two-layered graphene/Cu composite with graphene defects).

Defects were inserted into the graphene layers of the two- and four-layered graphene/Cu composites to establish the effect of defects on DOS analysis. Figure 8 presents the results for model 5 (Figure 2), in which defects were inserted into graphene in the two-layered graphene/Cu composite. DOS value of point A ($E_{}-E_{f}=-3.55$) is 140.65 e/eV^−1^ and that of point B ($E_{}-E_{f}=0$) is 17 e/eV^−1^. Compared with the two-layered graphene/Cu composite in which no defect was inserted, point A is lower by about 22.85% (41.67 e/eV^−1^) and point B is lower by about 50.63% (17.44 e/eV^−1^). This implies that defects in graphene reduce DOS value and decrease electrical performance.

**Figure 9:**
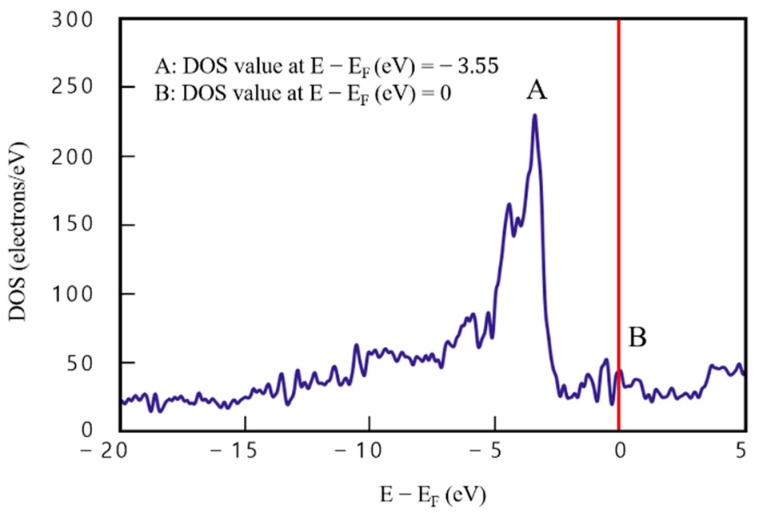
Density of states results for model 6 (four-layered graphene/Cu composite with graphene defects in the first layer).

Figure 9 presents DOS analysis results for model 6. Point A ($E_{}-E_{f}=-3.55$) corresponds to 201.87 e/eV^−1^ and point B ($E_{}-E_{f}=0$) to 44.2 e/eV^−1^. Compared with the four-layered graphene/Cu composite without defects, point A is lower by 7.25 e/eV^−1^ (about 3.47%) and point B is higher by 2.74 e/eV^−1^ (about 6.61%). Although defects in the external graphene layer (first layer) in the four-layered graphene/Cu composites led to a slight decrease in DOS value of point A, defects are nevertheless considered to play a doping-like role and increase electrical performance at point B.

**Figure 10:**
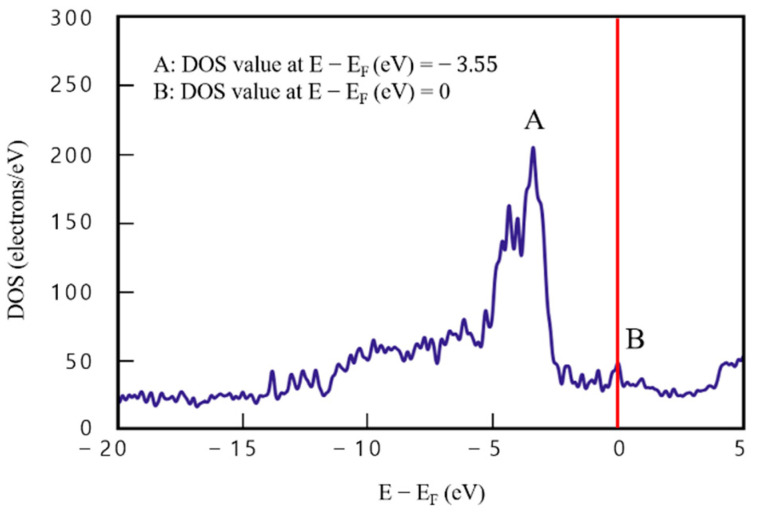
Density of states results for model 7 (four-layered graphene/Cu composite with graphene defects in the third layer).

To examine the effect of these defects in detail, DOS analysis of model 7 was performed by fixing the number of layers and changing only the defect location. Accordingly, the defects were inserted into the middle graphene layer (third layer) instead of the external graphene layer (first layer). The results are presented in Figure 10.

DOS analysis results of model 7 are presented in Figure 10. Point A ($E_{}-E_{f}=-3.55$) corresponds to 179.94 e/eV^−1^ and point B ($E_{}-E_{f}=0$) to 47.77 e/eV^−1^. Compared with Figure 7, which presents DOS findings of the defect-free four-layered Cu/graphene composite, Figure 10 shows that point A is lower by 29.18 e/eV^−1^ (about 13.95%), and point B by 6.31 e/eV^−1^ (about 15.22%). In the four-layered graphene/Cu composites, defects in the middle graphene layer (third layer) led to a slight decrease in DOS value of point A, similar to the effect of defects in the external graphene layer (first layer). However, DOS of point B indicates that defects play a doping-like role, facilitating electron movement and thereby improving electrical performance.

**Figure 11:**
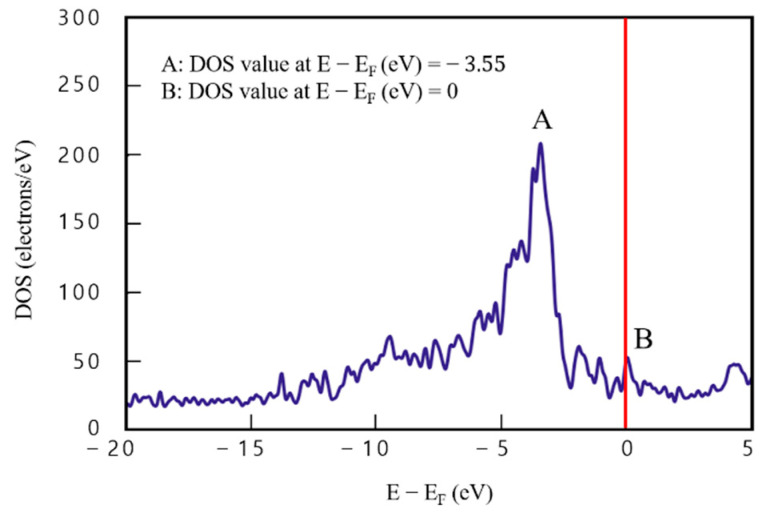
Density of states results for model 8 (four-layered graphene/Cu composite with graphene defects in the first and third layers).

The behavior of points A and B in DOS analyses was examined in detail using model 8 (Figure 3c). In this model, defects were inserted into both the external graphene layer (first layer) and middle graphene layer (third layer) of the four-layered graphene/Cu composite. DOS results are presented in Figure 11.

DOS analysis results of model 8 are presented in Figure 11. Point A ($E_{}-E_{f}=-3.55$) corresponds to 208 e/eV^−1^ and point B ($E_{}-E_{f}=0$) to 52 e/eV^−1^. Figure 9, Figure 10 and Figure 11 display similar trends. In Figure 11, point A is lower by about 0.05%, and point B is higher by about 25.42%, compared with the defect-free four-layered graphene/Cu composite. Defects in both the external graphene layer (first layer) and middle graphene layer (third layer) in the four-layered graphene/Cu composite led to a slight decrease in DOS value of the maximum (point A), but these defects are considered to improve electrical performance. Overall, the results indicate that defects have a significant positive effect on the electrical performance of graphene/Cu composites at point B of DOS, where the energy level is 0, and that DOS trends higher.

### 3.4. Effects of Defects in Charge Density of Graphene/Cu Composites

**Figure 12:**
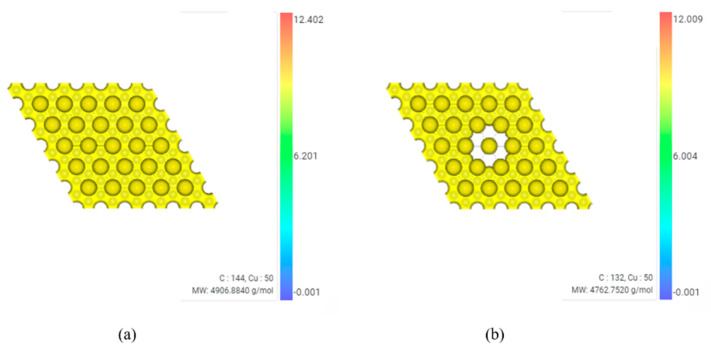
Charge density. (**a**) model 5 (four-layered graphene/Cu) (**b**) model 8 (four-layered graphene/Cu composite with graphene defects in the first and third layers).

Charge density of model 8 with graphene defects in the first and third layers compared to that of model 5, four-layered graphene/Cu composite, is shown in Figure 12.

Molecular weight(MW) of model 5 is 4906.88 g/mol. MW of model 8 is 4762.75 g/mol. In model 5, the range of ρ(max) is 12.402 e/bohr^3^ and ρ(min) is −0.00053 e/bohr^3^. In model 8, the range of ρ(max) is 12.009 e/bohr^3^ and ρ(min) is −0.00056 e/bohr^3^. Therefore, it can be seen that the charge density decreases by about 3.167% based on ρ(max) due to defects. It is judged that this is because the existence of defects prevented the bonding force of electrons.

## 4. Conclusions

DOS analyses of graphene/Cu composites containing defects were conducted to estimate the effect of defects on electrical performance. The following conclusions were drawn:

- DOS behavior of the two-layered graphene/Cu composite (model 3) resembled that of a single graphene layer, i.e., a stable DOS value was observed across the entire section of $-20<E_{}-E_{f}<5$ for the composite and across $-3<E_{}-E_{f}<0$ for Cu. This indicated that DOS of the two-layered graphene/Cu composite was affected by both graphene and Cu.
- Compared with the two-layered case, DOS value of the four-layered graphene/Cu composite was higher by ≥14.69% at point A, having the highest DOS value. Notably, DOS values over the sections $-20<E_{}-E_{f}<-5$ and $3<E_{}-E_{f}<5$ were double, which would be expected to improve electrical performance.
- In the two-layered graphene/Cu composite with graphene defects, point A was lower by about 22.85%, and point B by 50.63%, compared with the defect-free two-layered graphene/Cu composite.
- In the four-layered graphene/Cu composite containing defects in the external graphene layer (first layer), DOS value at point A was about 3.47% lower than that of the defect-free four-layered graphene/Cu composite, while DOS value of point B was higher by about 6.61%. In the four-layered graphene/Cu composite containing defects in the middle graphene layer (third layer), point A was lower by about 13.95% and B was higher by about 15.22%. Therefore, it is likely that the presence of graphene defects in four-layered graphene/Cu composites decreases the number of electrons at point A, and plays a doping-like role to increase electrical performance at point B. In addition, the external graphene defects performed better than the middle graphene defects at point A, but not at point B.
- In the four-layered graphene/Cu composites containing defects both in the external graphene layer (first layer) and middle graphene layer (third layer), DOS value of point A was only about 0.05% lower than that for the defect-free four-layered graphene/Cu composite, while the value of point B was about 25.42% higher. This means that the graphene defects decreased the number of electrons at point A and played a doping-like role, facilitating movement of electrons at point B and thereby improving electrical performance.

This study presents basic data concerning the electrical behavior of graphene/Cu composites containing defects. Understanding the influence of such defects is essential for the commercialization of graphene, and to control the physical properties of graphene/Cu composites.

## Figures and Tables

**Table 1:** Types of pseudo potential and cell parameter used with Quantum Espresso software.

Element	Pseudo Potential Types	Cell Parameter (a, b, c) (Å)	Cell Parameter (α, β, γ) (°)
Cu	C.pbe-n-kjpaw_psl.1.0.0.UPF	14.77, 14.77, 24.5	90°, 90°, 119.98°
Graphene	Cu_pbe_v1.2.uspp.F.UPF	14.77, 14.77, 20	

**Table 2:** Density functional theory analysis models.

Model	Type	Thickness (Å)	The Number of Atom	Interlayer Distance (Å)	Lattice Parameter
1	Single layer of graphene	20	Carbon, C 72	2.06	27.9
2	Single layer of Cu	24.5	Cu 25		
3	Two-layered Cu/graphene composite	46.56	C 72, Cu 25		
4	Four-layered Cu/graphene composite	95.18	C 144, Cu 50		
5	Two-layered Cu/graphene composite containing graphene defects	46.56	C 66, Cu 25		
6	Four-layered Cu/graphene composite with a first layer containing graphene defects	95.18	C 138, Cu 50		
7	Four-layered Cu/graphene composite with a third layer containing graphene defects				
8	Four-layered Cu/graphene composite with first- and third-layers containing graphene defects		C 132, Cu 50		

## Data Availability

The data presented in this study are available on request from the corresponding author.

## Supplementary Materials

The following supporting information can be downloaded at: https://www.mdpi.com/article/10.3390/ma16030962/s1, File S1: The coordinates of Model 1 to 8.
