# Supplementary material for: Effect of Defects in Graphene/Cu Composites on the Density of States
Source: Materials (Basel). 2023 Jan 20;16(3):962. doi: 10.3390/ma16030962 (PMC9917802; doi:10.3390/ma16030962)
Supplement: Supplementary file 1 [file materials-16-00962-s001.zip › materials-1982325-supplementary.pdf]

Supporting information for the readers

(1) The coordinates of Model 1

C 1.2305 0.71042951 0

C 0 1.42085901 0

C 0 2.84329617 0

C -1.2305 3.55372568 0

C -1.2305 4.97616284 0

C -2.461 5.68659235 0

C -2.461 7.10902951 0

C -3.6915 7.81945901 0

C -3.6915 9.24189617 0

C -4.922 9.95232568 0

C -4.922 11.37476284 0

C -6.1525 12.08519235 0

C 3.6915 0.71042951 0

C 2.461 1.42085901 0

C 2.461 2.84329617 0

C 1.2305 3.55372568 0

C 1.2305 4.97616284 0

C 0 5.68659235 0

C 0 7.10902951 0

C -1.2305 7.81945901 0

C -1.2305 9.24189617 0

C -2.461 9.95232568 0

C -2.461 11.37476284 0

C -3.6915 12.08519235 0

C 6.1525 0.71042951 0

C 4.922 1.42085901 0

C 4.922 2.84329617 0

C 3.6915 3.55372568 0

C 3.6915 4.97616284 0

C 2.461 5.68659235 0

C 2.461 7.10902951 0

C 1.2305 7.81945901 0

C 1.2305 9.24189617 0

C 0 9.95232568 0

C 0 11.37476284 0

C -1.2305 12.08519235 0

C 8.6135 0.71042951 0

C 7.383 1.42085901 0

C 7.383 2.84329617 0

C 6.1525 3.55372568 0

C 6.1525 4.97616284 0

C 4.922 5.68659235 0

C 4.922 7.10902951 0

C 3.6915 7.81945901 0

C 3.6915 9.24189617 0

C 2.461 9.95232568 0

C 2.461 11.37476284 0

C 1.2305 12.08519235 0

C 11.0745 0.71042951 0

C 9.844 1.42085901 0

C 9.844 2.84329617 0

C 8.6135 3.55372568 0

C 8.6135 4.97616284 0

C 7.383 5.68659235 0

C 7.383 7.10902951 0

C 6.1525 7.81945901 0

C 6.1525 9.24189617 0

C 4.922 9.95232568 0

C 4.922 11.37476284 0

C 3.6915 12.08519235 0

C 13.5355 0.71042951 0

C 12.305 1.42085901 0

C 12.305 2.84329617 0

C 11.0745 3.55372568 0

C 11.0745 4.97616284 0

C 9.844 5.68659235 0

C 9.844 7.10902951 0

C 8.6135 7.81945901 0

C 8.6135 9.24189617 0

C 7.383 9.95232568 0

C 7.383 11.37476284 0

C 6.1525 12.08519235 0

(2) The coordinates of Model 2

Cu 0 4.26494426 4.5

Cu 2.461 4.26494426 4.5

Cu 1.2305 2.13207759 4.5

Cu 3.6915 2.13207759 4.5

Cu 6.1525 2.13207759 4.5

Cu 4.922 4.26494426 4.5

Cu -3.6915 10.66354426 4.5

Cu -1.2305 10.66354426 4.5

Cu -2.461 8.53067759 4.5

Cu 0 8.53067759 4.5

Cu 2.461 8.53067759 4.5

Cu 1.2305 10.66354426 4.5

Cu 7.383 4.26494426 4.5

Cu 9.844 4.26494426 4.5

Cu 8.6135 2.13207759 4.5

Cu 11.0745 2.13207759 4.5

Cu 3.6915 10.66354426 4.5

Cu 6.1525 10.66354426 4.5

Cu 4.922 8.53067759 4.5

Cu 7.383 8.53067759 4.5

Cu -1.2305 6.39781092 4.5

Cu 1.2305 6.39781092 4.5

Cu 3.6912 6.39781092 4.5

Cu 6.1522 6.39781092 4.5

Cu 8.6132 6.39781092 4.5

(3) The coordinates of Model 3

C 1.2305 0.71042951 3

C 0 1.42085901 3

C 0 2.84329617 3

C -1.2305 3.55372568 3

C -1.2305 4.97616284 3

C -2.461 5.68659235 3

C 3.6915 0.71042951 3

C 2.461 1.42085901 3

C 2.461 2.84329617 3

C 1.2305 3.55372568 3

C 1.2305 4.97616284 3

C 0 5.68659235 3

C 6.1525 0.71042951 3

C 4.922 1.42085901 3

C 4.922 2.84329617 3

C 3.6915 3.55372568 3

C 3.6915 4.97616284 3

C 2.461 5.68659235 3

Cu 0 4.26494426 4.5

Cu 2.461 4.26494426 4.5

Cu 1.2305 2.13207759 4.5

Cu 3.6915 2.13207759 4.5

Cu 6.1525 2.13207759 4.5

Cu 4.922 4.26494426 4.5

C -2.461 7.10902951 3

C -3.6915 7.81945901 3

C -3.6915 9.24189617 3

C -4.922 9.95232568 3

C -4.922 11.37476284 3

C -6.1525 12.08519235 3

C 0 7.10902951 3

C -1.2305 7.81945901 3

C -1.2305 9.24189617 3

C -2.461 9.95232568 3

C -2.461 11.37476284 3

C -3.6915 12.08519235 3

C 2.461 7.10902951 3

C 1.2305 7.81945901 3

C 1.2305 9.24189617 3

C 0 9.95232568 3

C 0 11.37476284 3

C -1.2305 12.08519235 3

Cu -3.6915 10.66354426 4.5

Cu -1.2305 10.66354426 4.5

Cu -2.461 8.53067759 4.5

Cu 0 8.53067759 4.5

Cu 2.461 8.53067759 4.5

Cu 1.2305 10.66354426 4.5

C 8.6135 0.71042951 3

C 7.383 1.42085901 3

C 7.383 2.84329617 3

C 6.1525 3.55372568 3

C 6.1525 4.97616284 3

C 4.922 5.68659235 3

C 11.0745 0.71042951 3

C 9.844 1.42085901 3

C 9.844 2.84329617 3

C 8.6135 3.55372568 3

C 8.6135 4.97616284 3

C 7.383 5.68659235 3

C 13.5355 0.71042951 3

C 12.305 1.42085901 3

C 12.305 2.84329617 3

C 11.0745 3.55372568 3

C 11.0745 4.97616284 3

C 9.844 5.68659235 3

Cu 7.383 4.26494426 4.5

Cu 9.844 4.26494426 4.5

Cu 8.6135 2.13207759 4.5

Cu 11.0745 2.13207759 4.5

C 4.922 7.10902951 3

C 3.6915 7.81945901 3

C 3.6915 9.24189617 3

C 2.461 9.95232568 3

C 2.461 11.37476284 3

C 1.2305 12.08519235 3

C 7.383 7.10902951 3

C 6.1525 7.81945901 3

C 6.1525 9.24189617 3

C 4.922 9.95232568 3

C 4.922 11.37476284 3

C 3.6915 12.08519235 3

C 9.844 7.10902951 3

C 8.6135 7.81945901 3

C 8.6135 9.24189617 3

C 7.383 9.95232568 3

C 7.383 11.37476284 3

C 6.1525 12.08519235 3

Cu 3.6915 10.66354426 4.5

Cu 6.1525 10.66354426 4.5

Cu 4.922 8.53067759 4.5

Cu 7.383 8.53067759 4.5

Cu -1.2305 6.39781092 4.5

Cu 1.2305 6.39781092 4.5

Cu 3.6912 6.39781092 4.5

Cu 6.1522 6.39781092 4.5

Cu 8.6132 6.39781092 4.5

(4) The coordinates of Model 4

C 1.2305 0.71042951 0

C 0 1.42085901 0

C 0 2.84329617 0

C -1.2305 3.55372568 0

C -1.2305 4.97616284 0

C -2.461 5.68659235 0

C 3.6915 0.71042951 0

C 2.461 1.42085901 0

C 2.461 2.84329617 0

C 1.2305 3.55372568 0

C 1.2305 4.97616284 0

C 0 5.68659235 0

C 6.1525 0.71042951 0

C 4.922 1.42085901 0

C 4.922 2.84329617 0

C 3.6915 3.55372568 0

C 3.6915 4.97616284 0

C 2.461 5.68659235 0

Cu 0 4.26494426 1.5

Cu 2.461 4.26494426 1.5

Cu 1.2305 2.13207759 1.5

Cu 3.6915 2.13207759 1.5

C 1.2305 0.71042951 3

C 0 1.42085901 3

C 0 2.84329617 3

C -1.2305 3.55372568 3

C -1.2305 4.97616284 3

C -2.461 5.68659235 3

C 3.6915 0.71042951 3

C 2.461 1.42085901 3

C 2.461 2.84329617 3

C 1.2305 3.55372568 3

C 1.2305 4.97616284 3

C 0 5.68659235 3

C 6.1525 0.71042951 3

C 4.922 1.42085901 3

C 4.922 2.84329617 3

C 3.6915 3.55372568 3

C 3.6915 4.97616284 3

C 2.461 5.68659235 3

Cu 0 4.26494426 4.5

Cu 2.461 4.26494426 4.5

Cu 1.2305 2.13207759 4.5

Cu 3.6915 2.13207759 4.5

Cu 6.1525 2.13207759 4.5

Cu 4.922 4.26494426 4.5

Cu 6.1525 2.13207759 1.5

Cu 4.922 4.26494426 1.5

C -2.461 7.10902951 0

C -3.6915 7.81945901 0

C -3.6915 9.24189617 0

C -4.922 9.95232568 0

C -4.922 11.37476284 0

C -6.1525 12.08519235 0

C 0 7.10902951 0

C -1.2305 7.81945901 0

C -1.2305 9.24189617 0

C -2.461 9.95232568 0

C -2.461 11.37476284 0

C -3.6915 12.08519235 0

C 2.461 7.10902951 0

C 1.2305 7.81945901 0

C 1.2305 9.24189617 0

C 0 9.95232568 0

C 0 11.37476284 0

C -1.2305 12.08519235 0

Cu -3.6915 10.66354426 1.5

Cu -1.2305 10.66354426 1.5

Cu -2.461 8.53067759 1.5

Cu 0 8.53067759 1.5

C -2.461 7.10902951 3

C -3.6915 7.81945901 3

C -3.6915 9.24189617 3

C -4.922 9.95232568 3

C -4.922 11.37476284 3

C -6.1525 12.08519235 3

C 0 7.10902951 3

C -1.2305 7.81945901 3

C -1.2305 9.24189617 3

C -2.461 9.95232568 3

C -2.461 11.37476284 3

C -3.6915 12.08519235 3

C 2.461 7.10902951 3

C 1.2305 7.81945901 3

C 1.2305 9.24189617 3

C 0 9.95232568 3

C 0 11.37476284 3

C -1.2305 12.08519235 3

Cu -3.6915 10.66354426 4.5

Cu -1.2305 10.66354426 4.5

Cu -2.461 8.53067759 4.5

Cu 0 8.53067759 4.5

Cu 2.461 8.53067759 4.5

Cu 1.2305 10.66354426 4.5

Cu 2.461 8.53067759 1.5

Cu 1.2305 10.66354426 1.5

C 8.6135 0.71042951 0

C 7.383 1.42085901 0

C 7.383 2.84329617 0

C 6.1525 3.55372568 0

C 6.1525 4.97616284 0

C 4.922 5.68659235 0

C 11.0745 0.71042951 0

C 9.844 1.42085901 0

C 9.844 2.84329617 0

C 8.6135 3.55372568 0

C 8.6135 4.97616284 0

C 7.383 5.68659235 0

C 13.5355 0.71042951 0

C 12.305 1.42085901 0

C 12.305 2.84329617 0

C 11.0745 3.55372568 0

C 11.0745 4.97616284 0

C 9.844 5.68659235 0

Cu 7.383 4.26494426 1.5

Cu 9.844 4.26494426 1.5

Cu 8.6135 2.13207759 1.5

Cu 11.0745 2.13207759 1.5

C 8.6135 0.71042951 3

C 7.383 1.42085901 3

C 7.383 2.84329617 3

C 6.1525 3.55372568 3

C 6.1525 4.97616284 3

C 4.922 5.68659235 3

C 11.0745 0.71042951 3

C 9.844 1.42085901 3

C 9.844 2.84329617 3

C 8.6135 3.55372568 3

C 8.6135 4.97616284 3

C 7.383 5.68659235 3

C 13.5355 0.71042951 3

C 12.305 1.42085901 3

C 12.305 2.84329617 3

C 11.0745 3.55372568 3

C 11.0745 4.97616284 3

C 9.844 5.68659235 3

Cu 7.383 4.26494426 4.5

Cu 9.844 4.26494426 4.5

Cu 8.6135 2.13207759 4.5

Cu 11.0745 2.13207759 4.5

C 4.922 7.10902951 0

C 3.6915 7.81945901 0

C 3.6915 9.24189617 0

C 2.461 9.95232568 0

C 2.461 11.37476284 0

C 1.2305 12.08519235 0

C 7.383 7.10902951 0

C 6.1525 7.81945901 0

C 6.1525 9.24189617 0

C 4.922 9.95232568 0

C 4.922 11.37476284 0

C 3.6915 12.08519235 0

C 9.844 7.10902951 0

C 8.6135 7.81945901 0

C 8.6135 9.24189617 0

C 7.383 9.95232568 0

C 7.383 11.37476284 0

C 6.1525 12.08519235 0

Cu 3.6915 10.66354426 1.5

Cu 6.1525 10.66354426 1.5

Cu 4.922 8.53067759 1.5

Cu 7.383 8.53067759 1.5

C 4.922 7.10902951 3

C 3.6915 7.81945901 3

C 3.6915 9.24189617 3

C 2.461 9.95232568 3

C 2.461 11.37476284 3

C 1.2305 12.08519235 3

C 7.383 7.10902951 3

C 6.1525 7.81945901 3

C 6.1525 9.24189617 3

C 4.922 9.95232568 3

C 4.922 11.37476284 3

C 3.6915 12.08519235 3

C 9.844 7.10902951 3

C 8.6135 7.81945901 3

C 8.6135 9.24189617 3

C 7.383 9.95232568 3

C 7.383 11.37476284 3

C 6.1525 12.08519235 3

Cu 3.6915 10.66354426 4.5

Cu 6.1525 10.66354426 4.5

Cu 4.922 8.53067759 4.5

Cu 7.383 8.53067759 4.5

Cu -1.2305 6.39781092 4.5

Cu 1.2305 6.39781092 4.5

Cu 3.6912 6.39781092 4.5

Cu 6.1522 6.39781092 4.5

Cu 8.6132 6.39781092 4.5

Cu -1.2305 6.39781092 1.5

Cu 1.2305 6.39781092 1.5

Cu 3.6912 6.39781092 1.5

Cu 6.1522 6.39781092 1.5

Cu 8.6132 6.39781092 1.5

(5) The coordinates of Model 5

C 1.2305 0.71042951 0

C 0 1.42085901 0

C 0 2.84329617 0

C -1.2305 3.55372568 0

C -1.2305 4.97616284 0

C -2.461 5.68659235 0

C 3.6915 0.71042951 0

C 2.461 1.42085901 0

C 2.461 2.84329617 0

C 1.2305 3.55372568 0

C 1.2305 4.97616284 0

C 0 5.68659235 0

C 6.1525 0.71042951 0

C 4.922 1.42085901 0

C 4.922 2.84329617 0

C 3.6915 3.55372568 0

C 3.6915 4.97616284 0

C 2.461 5.68659235 0

Cu 0 4.26494426 1.5

Cu 2.461 4.26494426 1.5

Cu 1.2305 2.13207759 1.5

Cu 3.6915 2.13207759 1.5

C 1.2305 0.71042951 3

C 0 1.42085901 3

C 0 2.84329617 3

C -1.2305 3.55372568 3

C -1.2305 4.97616284 3

C -2.461 5.68659235 3

C 3.6915 0.71042951 3

C 2.461 1.42085901 3

C 2.461 2.84329617 3

C 1.2305 3.55372568 3

C 1.2305 4.97616284 3

C 0 5.68659235 3

C 6.1525 0.71042951 3

C 4.922 1.42085901 3

C 4.922 2.84329617 3

C 3.6915 3.55372568 3

C 3.6915 4.97616284 3

C 2.461 5.68659235 3

Cu 0 4.26494426 4.5

Cu 2.461 4.26494426 4.5

Cu 1.2305 2.13207759 4.5

Cu 3.6915 2.13207759 4.5

Cu 6.1525 2.13207759 4.5

Cu 4.922 4.26494426 4.5

Cu 6.1525 2.13207759 1.5

Cu 4.922 4.26494426 1.5

C -2.461 7.10902951 0

C -3.6915 7.81945901 0

C -3.6915 9.24189617 0

C -4.922 9.95232568 0

C -4.922 11.37476284 0

C -6.1525 12.08519235 0

C 0 7.10902951 0

C -1.2305 7.81945901 0

C -1.2305 9.24189617 0

C -2.461 9.95232568 0

C -2.461 11.37476284 0

C -3.6915 12.08519235 0

C 2.461 7.10902951 0

C 1.2305 7.81945901 0

C 1.2305 9.24189617 0

C 0 9.95232568 0

C 0 11.37476284 0

C -1.2305 12.08519235 0

Cu -3.6915 10.66354426 1.5

Cu -1.2305 10.66354426 1.5

Cu -2.461 8.53067759 1.5

Cu 0 8.53067759 1.5

C -2.461 7.10902951 3

C -3.6915 7.81945901 3

C -3.6915 9.24189617 3

C -4.922 9.95232568 3

C -4.922 11.37476284 3

C -6.1525 12.08519235 3

C 0 7.10902951 3

C -1.2305 7.81945901 3

C -1.2305 9.24189617 3

C -2.461 9.95232568 3

C -2.461 11.37476284 3

C -3.6915 12.08519235 3

C 2.461 7.10902951 3

C 1.2305 7.81945901 3

C 1.2305 9.24189617 3

C 0 9.95232568 3

C 0 11.37476284 3

C -1.2305 12.08519235 3

Cu -3.6915 10.66354426 4.5

Cu -1.2305 10.66354426 4.5

Cu -2.461 8.53067759 4.5

Cu 0 8.53067759 4.5

Cu 2.461 8.53067759 4.5

Cu 1.2305 10.66354426 4.5

Cu 2.461 8.53067759 1.5

Cu 1.2305 10.66354426 1.5

C 8.6135 0.71042951 0

C 7.383 1.42085901 0

C 7.383 2.84329617 0

C 6.1525 3.55372568 0

C 6.1525 4.97616284 0

C 4.922 5.68659235 0

C 11.0745 0.71042951 0

C 9.844 1.42085901 0

C 9.844 2.84329617 0

C 8.6135 3.55372568 0

C 8.6135 4.97616284 0

C 7.383 5.68659235 0

C 13.5355 0.71042951 0

C 12.305 1.42085901 0

C 12.305 2.84329617 0

C 11.0745 3.55372568 0

C 11.0745 4.97616284 0

C 9.844 5.68659235 0

Cu 7.383 4.26494426 1.5

Cu 9.844 4.26494426 1.5

Cu 8.6135 2.13207759 1.5

Cu 11.0745 2.13207759 1.5

C 8.6135 0.71042951 3

C 7.383 1.42085901 3

C 7.383 2.84329617 3

C 6.1525 3.55372568 3

C 6.1525 4.97616284 3

C 4.922 5.68659235 3

C 11.0745 0.71042951 3

C 9.844 1.42085901 3

C 9.844 2.84329617 3

C 8.6135 3.55372568 3

C 8.6135 4.97616284 3

C 7.383 5.68659235 3

C 13.5355 0.71042951 3

C 12.305 1.42085901 3

C 12.305 2.84329617 3

C 11.0745 3.55372568 3

C 11.0745 4.97616284 3

C 9.844 5.68659235 3

Cu 7.383 4.26494426 4.5

Cu 9.844 4.26494426 4.5

Cu 8.6135 2.13207759 4.5

Cu 11.0745 2.13207759 4.5

C 4.922 7.10902951 0

C 3.6915 7.81945901 0

C 3.6915 9.24189617 0

C 2.461 9.95232568 0

C 2.461 11.37476284 0

C 1.2305 12.08519235 0

C 7.383 7.10902951 0

C 6.1525 7.81945901 0

C 6.1525 9.24189617 0

C 4.922 9.95232568 0

C 4.922 11.37476284 0

C 3.6915 12.08519235 0

C 9.844 7.10902951 0

C 8.6135 7.81945901 0

C 8.6135 9.24189617 0

C 7.383 9.95232568 0

C 7.383 11.37476284 0

C 6.1525 12.08519235 0

Cu 3.6915 10.66354426 1.5

Cu 6.1525 10.66354426 1.5

Cu 4.922 8.53067759 1.5

Cu 7.383 8.53067759 1.5

C 4.922 7.10902951 3

C 3.6915 7.81945901 3

C 3.6915 9.24189617 3

C 2.461 9.95232568 3

C 2.461 11.37476284 3

C 1.2305 12.08519235 3

C 7.383 7.10902951 3

C 6.1525 7.81945901 3

C 6.1525 9.24189617 3

C 4.922 9.95232568 3

C 4.922 11.37476284 3

C 3.6915 12.08519235 3

C 9.844 7.10902951 3

C 8.6135 7.81945901 3

C 8.6135 9.24189617 3

C 7.383 9.95232568 3

C 7.383 11.37476284 3

C 6.1525 12.08519235 3

Cu 3.6915 10.66354426 4.5

Cu 6.1525 10.66354426 4.5

Cu 4.922 8.53067759 4.5

Cu 7.383 8.53067759 4.5

Cu -1.2305 6.39781092 4.5

Cu 1.2305 6.39781092 4.5

Cu 3.6912 6.39781092 4.5

Cu 6.1522 6.39781092 4.5

Cu 8.6132 6.39781092 4.5

Cu -1.2305 6.39781092 1.5

Cu 1.2305 6.39781092 1.5

Cu 3.6912 6.39781092 1.5

Cu 6.1522 6.39781092 1.5

Cu 8.6132 6.39781092 1.5

(6) The coordinates of Model 6

C 1.2305 0.71042951 0

C 0 1.42085901 0

C 0 2.84329617 0

C -1.2305 3.55372568 0

C -1.2305 4.97616284 0

C -2.461 5.68659235 0

C 3.6915 0.71042951 0

C 2.461 1.42085901 0

C 2.461 2.84329617 0

C 1.2305 3.55372568 0

C 1.2305 4.97616284 0

C 0 5.68659235 0

C 6.1525 0.71042951 0

C 4.922 1.42085901 0

C 4.922 2.84329617 0

C 3.6915 3.55372568 0

Cu 0 4.26494426 1.5

Cu 2.461 4.26494426 1.5

Cu 1.2305 2.13207759 1.5

Cu 3.6915 2.13207759 1.5

C 1.2305 0.71042951 3

C 0 1.42085901 3

C 0 2.84329617 3

C -1.2305 3.55372568 3

C -1.2305 4.97616284 3

C -2.461 5.68659235 3

C 3.6915 0.71042951 3

C 2.461 1.42085901 3

C 2.461 2.84329617 3

C 1.2305 3.55372568 3

C 1.2305 4.97616284 3

C 0 5.68659235 3

C 6.1525 0.71042951 3

C 4.922 1.42085901 3

C 4.922 2.84329617 3

C 3.6915 3.55372568 3

C 3.6915 4.97616284 3

C 2.461 5.68659235 3

Cu 0 4.26494426 4.5

Cu 2.461 4.26494426 4.5

Cu 1.2305 2.13207759 4.5

Cu 3.6915 2.13207759 4.5

Cu 6.1525 2.13207759 4.5

Cu 4.922 4.26494426 4.5

Cu 6.1525 2.13207759 1.5

Cu 4.922 4.26494426 1.5

C -2.461 7.10902951 0

C -3.6915 7.81945901 0

C -3.6915 9.24189617 0

C -4.922 9.95232568 0

C -4.922 11.37476284 0

C -6.1525 12.08519235 0

C 0 7.10902951 0

C -1.2305 7.81945901 0

C -1.2305 9.24189617 0

C -2.461 9.95232568 0

C -2.461 11.37476284 0

C -3.6915 12.08519235 0

C 1.2305 7.81945901 0

C 1.2305 9.24189617 0

C 0 9.95232568 0

C 0 11.37476284 0

C -1.2305 12.08519235 0

Cu -3.6915 10.66354426 1.5

Cu -1.2305 10.66354426 1.5

Cu -2.461 8.53067759 1.5

Cu 0 8.53067759 1.5

C -2.461 7.10902951 3

C -3.6915 7.81945901 3

C -3.6915 9.24189617 3

C -4.922 9.95232568 3

C -4.922 11.37476284 3

C -6.1525 12.08519235 3

C 0 7.10902951 3

C -1.2305 7.81945901 3

C -1.2305 9.24189617 3

C -2.461 9.95232568 3

C -2.461 11.37476284 3

C -3.6915 12.08519235 3

C 2.461 7.10902951 3

C 1.2305 7.81945901 3

C 1.2305 9.24189617 3

C 0 9.95232568 3

C 0 11.37476284 3

C -1.2305 12.08519235 3

Cu -3.6915 10.66354426 4.5

Cu -1.2305 10.66354426 4.5

Cu -2.461 8.53067759 4.5

Cu 0 8.53067759 4.5

Cu 2.461 8.53067759 4.5

Cu 1.2305 10.66354426 4.5

Cu 2.461 8.53067759 1.5

Cu 1.2305 10.66354426 1.5

C 8.6135 0.71042951 0

C 7.383 1.42085901 0

C 7.383 2.84329617 0

C 6.1525 3.55372568 0

C 6.1525 4.97616284 0

C 11.0745 0.71042951 0

C 9.844 1.42085901 0

C 9.844 2.84329617 0

C 8.6135 3.55372568 0

C 8.6135 4.97616284 0

C 7.383 5.68659235 0

C 13.5355 0.71042951 0

C 12.305 1.42085901 0

C 12.305 2.84329617 0

C 11.0745 3.55372568 0

C 11.0745 4.97616284 0

C 9.844 5.68659235 0

Cu 7.383 4.26494426 1.5

Cu 9.844 4.26494426 1.5

Cu 8.6135 2.13207759 1.5

Cu 11.0745 2.13207759 1.5

C 8.6135 0.71042951 3

C 7.383 1.42085901 3

C 7.383 2.84329617 3

C 6.1525 3.55372568 3

C 6.1525 4.97616284 3

C 4.922 5.68659235 3

C 11.0745 0.71042951 3

C 9.844 1.42085901 3

C 9.844 2.84329617 3

C 8.6135 3.55372568 3

C 8.6135 4.97616284 3

C 7.383 5.68659235 3

C 13.5355 0.71042951 3

C 12.305 1.42085901 3

C 12.305 2.84329617 3

C 11.0745 3.55372568 3

C 11.0745 4.97616284 3

C 9.844 5.68659235 3

Cu 7.383 4.26494426 4.5

Cu 9.844 4.26494426 4.5

Cu 8.6135 2.13207759 4.5

Cu 11.0745 2.13207759 4.5

C 3.6915 9.24189617 0

C 2.461 9.95232568 0

C 2.461 11.37476284 0

C 1.2305 12.08519235 0

C 7.383 7.10902951 0

C 6.1525 7.81945901 0

C 6.1525 9.24189617 0

C 4.922 9.95232568 0

C 4.922 11.37476284 0

C 3.6915 12.08519235 0

C 9.844 7.10902951 0

C 8.6135 7.81945901 0

C 8.6135 9.24189617 0

C 7.383 9.95232568 0

C 7.383 11.37476284 0

C 6.1525 12.08519235 0

Cu 3.6915 10.66354426 1.5

Cu 6.1525 10.66354426 1.5

Cu 4.922 8.53067759 1.5

Cu 7.383 8.53067759 1.5

C 4.922 7.10902951 3

C 3.6915 7.81945901 3

C 3.6915 9.24189617 3

C 2.461 9.95232568 3

C 2.461 11.37476284 3

C 1.2305 12.08519235 3

C 7.383 7.10902951 3

C 6.1525 7.81945901 3

C 6.1525 9.24189617 3

C 4.922 9.95232568 3

C 4.922 11.37476284 3

C 3.6915 12.08519235 3

C 9.844 7.10902951 3

C 8.6135 7.81945901 3

C 8.6135 9.24189617 3

C 7.383 9.95232568 3

C 7.383 11.37476284 3

C 6.1525 12.08519235 3

Cu 3.6915 10.66354426 4.5

Cu 6.1525 10.66354426 4.5

Cu 4.922 8.53067759 4.5

Cu 7.383 8.53067759 4.5

Cu -1.2305 6.39781092 4.5

Cu 1.2305 6.39781092 4.5

Cu 3.6912 6.39781092 4.5

Cu 6.1522 6.39781092 4.5

Cu 8.6132 6.39781092 4.5

Cu -1.2305 6.39781092 1.5

Cu 1.2305 6.39781092 1.5

Cu 3.6912 6.39781092 1.5

Cu 6.1522 6.39781092 1.5

Cu 8.6132 6.39781092 1.5

(7) The coordinates of Model 7

C 1.2305 0.71042951 0

C 0 1.42085901 0

C 0 2.84329617 0

C -1.2305 3.55372568 0

C -1.2305 4.97616284 0

C -2.461 5.68659235 0

C 3.6915 0.71042951 0

C 2.461 1.42085901 0

C 2.461 2.84329617 0

C 1.2305 3.55372568 0

C 1.2305 4.97616284 0

C 0 5.68659235 0

C 6.1525 0.71042951 0

C 4.922 1.42085901 0

C 4.922 2.84329617 0

C 3.6915 3.55372568 0

C 3.6915 4.97616284 0

C 2.461 5.68659235 0

Cu 0 4.26494426 1.5

Cu 2.461 4.26494426 1.5

Cu 1.2305 2.13207759 1.5

Cu 3.6915 2.13207759 1.5

C 1.2305 0.71042951 3

C 0 1.42085901 3

C 0 2.84329617 3

C -1.2305 3.55372568 3

C -1.2305 4.97616284 3

C -2.461 5.68659235 3

C 3.6915 0.71042951 3

C 2.461 1.42085901 3

C 2.461 2.84329617 3

C 1.2305 3.55372568 3

C 1.2305 4.97616284 3

C 0 5.68659235 3

C 6.1525 0.71042951 3

C 4.922 1.42085901 3

C 4.922 2.84329617 3

C 3.6915 3.55372568 3

Cu 0 4.26494426 4.5

Cu 2.461 4.26494426 4.5

Cu 1.2305 2.13207759 4.5

Cu 3.6915 2.13207759 4.5

Cu 6.1525 2.13207759 4.5

Cu 4.922 4.26494426 4.5

Cu 6.1525 2.13207759 1.5

Cu 4.922 4.26494426 1.5

C -2.461 7.10902951 0

C -3.6915 7.81945901 0

C -3.6915 9.24189617 0

C -4.922 9.95232568 0

C -4.922 11.37476284 0

C -6.1525 12.08519235 0

C 0 7.10902951 0

C -1.2305 7.81945901 0

C -1.2305 9.24189617 0

C -2.461 9.95232568 0

C -2.461 11.37476284 0

C -3.6915 12.08519235 0

C 2.461 7.10902951 0

C 1.2305 7.81945901 0

C 1.2305 9.24189617 0

C 0 9.95232568 0

C 0 11.37476284 0

C -1.2305 12.08519235 0

Cu -3.6915 10.66354426 1.5

Cu -1.2305 10.66354426 1.5

Cu -2.461 8.53067759 1.5

Cu 0 8.53067759 1.5

C -2.461 7.10902951 3

C -3.6915 7.81945901 3

C -3.6915 9.24189617 3

C -4.922 9.95232568 3

C -4.922 11.37476284 3

C -6.1525 12.08519235 3

C 0 7.10902951 3

C -1.2305 7.81945901 3

C -1.2305 9.24189617 3

C -2.461 9.95232568 3

C -2.461 11.37476284 3

C -3.6915 12.08519235 3

C 1.2305 7.81945901 3

C 1.2305 9.24189617 3

C 0 9.95232568 3

C 0 11.37476284 3

C -1.2305 12.08519235 3

Cu -3.6915 10.66354426 4.5

Cu -1.2305 10.66354426 4.5

Cu -2.461 8.53067759 4.5

Cu 0 8.53067759 4.5

Cu 2.461 8.53067759 4.5

Cu 1.2305 10.66354426 4.5

Cu 2.461 8.53067759 1.5

Cu 1.2305 10.66354426 1.5

C 8.6135 0.71042951 0

C 7.383 1.42085901 0

C 7.383 2.84329617 0

C 6.1525 3.55372568 0

C 6.1525 4.97616284 0

C 4.922 5.68659235 0

C 11.0745 0.71042951 0

C 9.844 1.42085901 0

C 9.844 2.84329617 0

C 8.6135 3.55372568 0

C 8.6135 4.97616284 0

C 7.383 5.68659235 0

C 13.5355 0.71042951 0

C 12.305 1.42085901 0

C 12.305 2.84329617 0

C 11.0745 3.55372568 0

C 11.0745 4.97616284 0

C 9.844 5.68659235 0

Cu 7.383 4.26494426 1.5

Cu 9.844 4.26494426 1.5

Cu 8.6135 2.13207759 1.5

Cu 11.0745 2.13207759 1.5

C 8.6135 0.71042951 3

C 7.383 1.42085901 3

C 7.383 2.84329617 3

C 6.1525 3.55372568 3

C 6.1525 4.97616284 3

C 11.0745 0.71042951 3

C 9.844 1.42085901 3

C 9.844 2.84329617 3

C 8.6135 3.55372568 3

C 8.6135 4.97616284 3

C 7.383 5.68659235 3

C 13.5355 0.71042951 3

C 12.305 1.42085901 3

C 12.305 2.84329617 3

C 11.0745 3.55372568 3

C 11.0745 4.97616284 3

C 9.844 5.68659235 3

Cu 7.383 4.26494426 4.5

Cu 9.844 4.26494426 4.5

Cu 8.6135 2.13207759 4.5

Cu 11.0745 2.13207759 4.5

C 4.922 7.10902951 0

C 3.6915 7.81945901 0

C 3.6915 9.24189617 0

C 2.461 9.95232568 0

C 2.461 11.37476284 0

C 1.2305 12.08519235 0

C 7.383 7.10902951 0

C 6.1525 7.81945901 0

C 6.1525 9.24189617 0

C 4.922 9.95232568 0

C 4.922 11.37476284 0

C 3.6915 12.08519235 0

C 9.844 7.10902951 0

C 8.6135 7.81945901 0

C 8.6135 9.24189617 0

C 7.383 9.95232568 0

C 7.383 11.37476284 0

C 6.1525 12.08519235 0

Cu 3.6915 10.66354426 1.5

Cu 6.1525 10.66354426 1.5

Cu 4.922 8.53067759 1.5

Cu 7.383 8.53067759 1.5

C 3.6915 9.24189617 3

C 2.461 9.95232568 3

C 2.461 11.37476284 3

C 1.2305 12.08519235 3

C 7.383 7.10902951 3

C 6.1525 7.81945901 3

C 6.1525 9.24189617 3

C 4.922 9.95232568 3

C 4.922 11.37476284 3

C 3.6915 12.08519235 3

C 9.844 7.10902951 3

C 8.6135 7.81945901 3

C 8.6135 9.24189617 3

C 7.383 9.95232568 3

C 7.383 11.37476284 3

C 6.1525 12.08519235 3

Cu 3.6915 10.66354426 4.5

Cu 6.1525 10.66354426 4.5

Cu 4.922 8.53067759 4.5

Cu 7.383 8.53067759 4.5

Cu -1.2305 6.39781092 4.5

Cu 1.2305 6.39781092 4.5

Cu 3.6912 6.39781092 4.5

Cu 6.1522 6.39781092 4.5

Cu 8.6132 6.39781092 4.5

Cu -1.2305 6.39781092 1.5

Cu 1.2305 6.39781092 1.5

Cu 3.6912 6.39781092 1.5

Cu 6.1522 6.39781092 1.5

Cu 8.6132 6.39781092 1.5

(8) The coordinates of Model 8

C 1.2305 0.71042951 3

C 0 1.42085901 3

C 0 2.84329617 3

C -1.2305 3.55372568 3

C -1.2305 4.97616284 3

C -2.461 5.68659235 3

C 3.6915 0.71042951 3

C 2.461 1.42085901 3

C 2.461 2.84329617 3

C 1.2305 3.55372568 3

C 1.2305 4.97616284 3

C 0 5.68659235 3

C 6.1525 0.71042951 3

C 4.922 1.42085901 3

C 4.922 2.84329617 3

C 3.6915 3.55372568 3

Cu 0 4.26494426 4.5

Cu 2.461 4.26494426 4.5

Cu 1.2305 2.13207759 4.5

Cu 3.6915 2.13207759 4.5

Cu 6.1525 2.13207759 4.5

Cu 4.922 4.26494426 4.5

C -2.461 7.10902951 3

C -3.6915 7.81945901 3

C -3.6915 9.24189617 3

C -4.922 9.95232568 3

C -4.922 11.37476284 3

C -6.1525 12.08519235 3

C 0 7.10902951 3

C -1.2305 7.81945901 3

C -1.2305 9.24189617 3

C -2.461 9.95232568 3

C -2.461 11.37476284 3

C -3.6915 12.08519235 3

C 1.2305 7.81945901 3

C 1.2305 9.24189617 3

C 0 9.95232568 3

C 0 11.37476284 3

C -1.2305 12.08519235 3

Cu -3.6915 10.66354426 4.5

Cu -1.2305 10.66354426 4.5

Cu -2.461 8.53067759 4.5

Cu 0 8.53067759 4.5

Cu 2.461 8.53067759 4.5

Cu 1.2305 10.66354426 4.5

C 8.6135 0.71042951 3

C 7.383 1.42085901 3

C 7.383 2.84329617 3

C 6.1525 3.55372568 3

C 6.1525 4.97616284 3

C 11.0745 0.71042951 3

C 9.844 1.42085901 3

C 9.844 2.84329617 3

C 8.6135 3.55372568 3

C 8.6135 4.97616284 3

C 7.383 5.68659235 3

C 13.5355 0.71042951 3

C 12.305 1.42085901 3

C 12.305 2.84329617 3

C 11.0745 3.55372568 3

C 11.0745 4.97616284 3

C 9.844 5.68659235 3

Cu 7.383 4.26494426 4.5

Cu 9.844 4.26494426 4.5

Cu 8.6135 2.13207759 4.5

Cu 11.0745 2.13207759 4.5

C 3.6915 9.24189617 3

C 2.461 9.95232568 3

C 2.461 11.37476284 3

C 1.2305 12.08519235 3

C 7.383 7.10902951 3

C 6.1525 7.81945901 3

C 6.1525 9.24189617 3

C 4.922 9.95232568 3

C 4.922 11.37476284 3

C 3.6915 12.08519235 3

C 9.844 7.10902951 3

C 8.6135 7.81945901 3

C 8.6135 9.24189617 3

C 7.383 9.95232568 3

C 7.383 11.37476284 3

C 6.1525 12.08519235 3

Cu 3.6915 10.66354426 4.5

Cu 6.1525 10.66354426 4.5

Cu 4.922 8.53067759 4.5

Cu 7.383 8.53067759 4.5

Cu -1.2305 6.39781092 4.5

Cu 1.2305 6.39781092 4.5

Cu 3.6912 6.39781092 4.5

Cu 6.1522 6.39781092 4.5

Cu 8.6132 6.39781092 4.5

C 1.2305 0.71042951 6

C 0 1.42085901 6

C 0 2.84329617 6

C -1.2305 3.55372568 6

C -1.2305 4.97616284 6

C -2.461 5.68659235 6

C 3.6915 0.71042951 6

C 2.461 1.42085901 6

C 2.461 2.84329617 6

C 1.2305 3.55372568 6

C 1.2305 4.97616284 6

C 0 5.68659235 6

C 6.1525 0.71042951 6

C 4.922 1.42085901 6

C 4.922 2.84329617 6

C 3.6915 3.55372568 6

Cu 0 4.26494426 7.5

Cu 2.461 4.26494426 7.5

Cu 1.2305 2.13207759 7.5

Cu 3.6915 2.13207759 7.5

Cu 6.1525 2.13207759 7.5

Cu 4.922 4.26494426 7.5

C -2.461 7.10902951 6

C -3.6915 7.81945901 6

C -3.6915 9.24189617 6

C -4.922 9.95232568 6

C -4.922 11.37476284 6

C -6.1525 12.08519235 6

C 0 7.10902951 6

C -1.2305 7.81945901 6

C -1.2305 9.24189617 6

C -2.461 9.95232568 6

C -2.461 11.37476284 6

C -3.6915 12.08519235 6

C 1.2305 7.81945901 6

C 1.2305 9.24189617 6

C 0 9.95232568 6

C 0 11.37476284 6

C -1.2305 12.08519235 6

Cu -3.6915 10.66354426 7.5

Cu -1.2305 10.66354426 7.5

Cu -2.461 8.53067759 7.5

Cu 0 8.53067759 7.5

Cu 2.461 8.53067759 7.5

Cu 1.2305 10.66354426 7.5

C 8.6135 0.71042951 6

C 7.383 1.42085901 6

C 7.383 2.84329617 6

C 6.1525 3.55372568 6

C 6.1525 4.97616284 6

C 11.0745 0.71042951 6

C 9.844 1.42085901 6

C 9.844 2.84329617 6

C 8.6135 3.55372568 6

C 8.6135 4.97616284 6

C 7.383 5.68659235 6

C 13.5355 0.71042951 6

C 12.305 1.42085901 6

C 12.305 2.84329617 6

C 11.0745 3.55372568 6

C 11.0745 4.97616284 6

C 9.844 5.68659235 6

Cu 7.383 4.26494426 7.5

Cu 9.844 4.26494426 7.5

Cu 8.6135 2.13207759 7.5

Cu 11.0745 2.13207759 7.5

C 3.6915 9.24189617 6

C 2.461 9.95232568 6

C 2.461 11.37476284 6

C 1.2305 12.08519235 6

C 7.383 7.10902951 6

C 6.1525 7.81945901 6

C 6.1525 9.24189617 6

C 4.922 9.95232568 6

C 4.922 11.37476284 6

C 3.6915 12.08519235 6

C 9.844 7.10902951 6

C 8.6135 7.81945901 6

C 8.6135 9.24189617 6

C 7.383 9.95232568 6

C 7.383 11.37476284 6

C 6.1525 12.08519235 6

Cu 3.6915 10.66354426 7.5

Cu 6.1525 10.66354426 7.5

Cu 4.922 8.53067759 7.5

Cu 7.383 8.53067759 7.5

Cu -1.2305 6.39781092 7.5

Cu 1.2305 6.39781092 7.5

Cu 3.6912 6.39781092 7.5

Cu 6.1522 6.39781092 7.5

Cu 8.6132 6.39781092 7.5
